# What remains from a 454 run: estimation of success rates of microsatellite loci development in selected newt species (*Calotriton asper, Lissotriton helveticus*, and *Triturus cristatus*) and comparison with Illumina-based approaches

**DOI:** 10.1002/ece3.764

**Published:** 2013-09-17

**Authors:** Axel Drechsler, Daniel Geller, Katharina Freund, Dirk S Schmeller, Sven Künzel, Oliver Rupp, Adeline Loyau, Mathieu Denoël, Emilio Valbuena-Ureña, Sebastian Steinfartz

**Affiliations:** 1 Department of Behavioral Biology, Unit of Molecular Ecology and Behavior, University of BielefeldMorgenbreede 45, D-33619, Bielefeld, Germany; 2 Helmholtz Center for Environmental Research – UFZPermoserstrasse 15, 04318, Leipzig, Germany; 3 EcoLab (Laboratoire Ecologie Fonctionnelle et Environnement), Université de Toulouse, UPS, INPT118 route de Narbonne, 31062, Toulouse, France; 4 EcoLab, CNRS31062, Toulouse, France; 5 Max Planck Institute for Evolutionary BiologyAugust-Thienemann-Str. 2, 24306, Plön, Germany; 6 Center for Biotechnology, University of BielefeldUniversitätsstraße 27, D-33615, Bielefeld, Germany; 7 Department of Biology, Ecology and Evolution, Laboratory of Fish and Amphibian Ethology, Behavioral Biology Unit, University of LiegeQuai van Beneden 22, 4020, Liege, Belgium; 8 Unitat de Zoologia, Facultat de Biociències, Universitat Autònoma de Barcelona08193, Cerdanyola del Vallès, Barcelona, Catalonia, Spain; 9 Centre de Fauna Salvatge de Torreferrussa (Catalan Wildlife Service- Forestal Catalana). Finca de Torreferrusa, Crta B-140Km 4,5. 08130, Santa Perpètua de la Mogoda, Barcelona, Spain

**Keywords:** Amphibians, *Calotriton arnoldi*, crested newt, cross-amplification success, genome size, PAL_FINDER, Pyrenean mountain newt

## Abstract

The development of microsatellite loci has become more efficient using next-generation sequencing (NGS) approaches, and many studies imply that the amount of applicable loci is large. However, few studies have sought to quantify the number of loci that are retained for use out of the thousands of sequence reads initially obtained. We analyzed the success rate of microsatellite loci development for three amphibian species using a 454 NGS approach on tetra-nucleotide motif-enriched species-specific libraries. The number of sequence reads obtained differed strongly between species and ranged from 19,562 for *Triturus cristatus* to 55,626 for *Lissotriton helveticus*, with 52,075 reads obtained for *Calotriton asper*. PHOBOS was used to identify sequences with tetra-nucleotide repeat motifs with a minimum repeat number of ten and high quality primer binding sites. Of 107 sequences for *T. cristatus*, 316 for *C. asper* and 319 for *L. helveticus,* we tested the amplification success, polymorphism, and degree of heterozygosity for 41 primer combinations each for *C. asper* and *T. cristatus,* and 22 for *L. helveticus*. We found 11 polymorphic loci for *T. cristatus*, 20 loci for *C. asper,* and 15 loci for *L. helveticus*. Extrapolated, the number of potentially amplifiable loci (PALs) resulted in estimated species-specific success rates of 0.15% (*T. cristatus*), 0.30% (*C. asper*), and 0.39% (*L. helveticus*). Compared with representative Illumina NGS approaches, our applied 454-sequencing approach on specifically enriched sublibraries proved to be quite competitive in terms of success rates and number of finally applicable loci.

## Introduction

Microsatellite loci are still considered valuable tools for addressing basic questions in ecology, evolution, and behavior in nonmodel organisms, despite the fact that other molecular markers have become increasingly popular due to next-generation sequencing (NGS) approaches (e.g., genotyping or sequencing of single-nucleotide polymorphisms (SNPs)). Microsatellite loci are currently still the marker of choice for comprehensive analyses of population structure (e.g., Palo et al. 2004; Jehle et al. 2005, 2007; Dogaç et al. 2013), mating systems (e.g., Jones et al. 2002; Schmeller et al. 2005; Steinfartz et al. 2006; Jehle et al. 2007; Loyau and Schmeller 2012), landscape (Storfer et al. 2010) and conservation genetics (reviewed by Jehle and Arntzen 2002; Beebee 2005; Schmeller and Merilä 2007; Duong et al. 2013). Moreover, the current impact of microsatellite loci as genetic tools is also illustrated by more than 4000 scientific studies that have been published in the past two years matching the query “microsatellite loci” in the Web of Science and by recent publications reporting the development of new loci (i.e., Prunier et al. 2012; Castoe et al. 2012a,b; Dobeš and Scheffknecht 2012). Before the application of NGS in the process of developing microsatellite loci, the isolation and characterization of new loci was a costly and sometimes elaborate endeavor (e.g., Zane et al. 2002). However, by using NGS approaches on genomic libraries enriched for microsatellite motifs, the isolation process has become much simpler and more cost-effective (e.g., Abdelkrim et al. 2009). Normally, these approaches result in tens of thousands of sequence reads, which are expected to lead to a large amount of suitable microsatellite loci (e.g., Yang et al. 2012). However, the correlation between the initial number of sequence reads obtained and the number of usable polymorphic microsatellite loci may be low as the number of potentially amplifiable loci (PALs) is negatively influenced by many factors. These factors include sequence read quality (cut-off score values), motif length (type and number of repeat), and the presence, quality, and necessary length of the primer region, in addition to the amplification success and confirmed polymorphism of loci across the studied populations. Accordingly, systematic approaches to estimate success rates of microsatellite loci development for quite distinct taxa are important to finally obtain a sufficient number of applicable loci (e.g., Castoe et al. 2010, 2012a,b; Prunier et al. 2012). For example, in the copperhead snake (*Agkistrodon contortrix*), Castoe et al. (2010), isolated 4,564 PALs from 128,773 reads, but only found 80 tetra-nucleotide PALs (i.e., 0.062% of all reads) with more than 10 repeat units. In the alpine newt (*Ichthyosaura alpestris*), Prunier et al. (2012) obtained 1015 microsatellite motif-bearing sequence reads, with a final yield of 14 microsatellite loci from 61 tested primer pair combinations. Microsatellite development might be especially tedious in amphibians due to their large genome sizes and comparably low numbers of PALs, which have made development and isolation approaches in the past both cost- and time-intensive (e.g., Hendrix et al. 2010; Hauswaldt et al. 2012). Accordingly, NGS-based microsatellite loci development approaches should be efficient in obtaining a sufficient number of loci in such species (e.g., amphibians).

In this study, we used a 454-sequencing approach with enriched libraries to develop highly polymorphic tetra-nucleotide microsatellite loci for three distinct newt species within the family of Salamandridae (*Calotriton asper, Lissotriton helveticus,* and *Triturus cristatus*). We determined the success rate of our approach by estimating the number of PALs based on the number of usable polymorphic loci tested across several populations of each species and compared it with Illumina-based sequencing approaches of recently published studies. Furthermore, we tested the cross-amplification success rate of the developed loci for *C. asper* in the highly endemic and threatened species *C. arnoldi,* the Montseny brook newt (see Carranza and Amat 2005).

## Materials and Methods

### Study species

**Figure 1:**
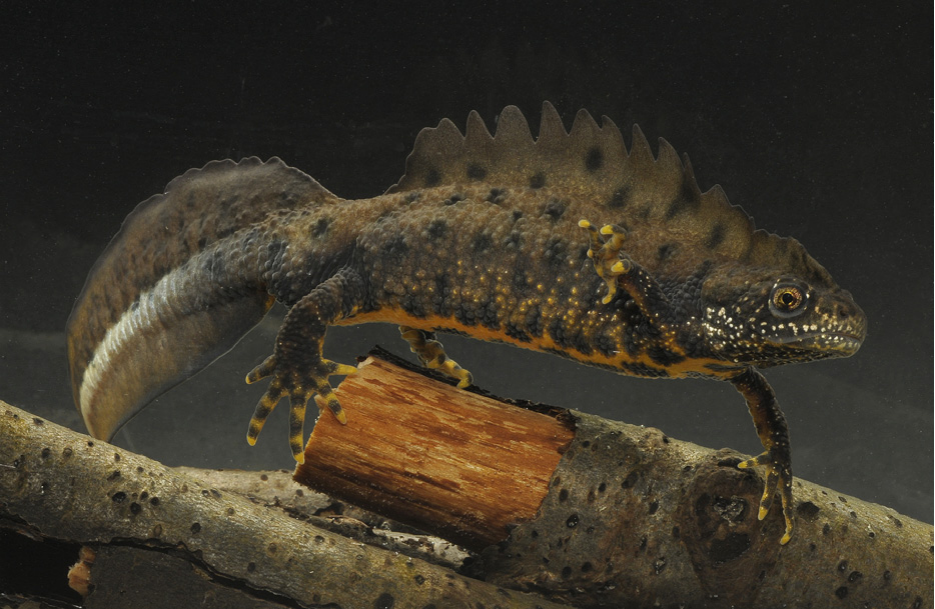
Male of the crested newt (*Triturus cristatus*), one of the target species for which microsatellite loci have been developed (photograph by B. Thiesmeier).

*Triturus cristatus*, the great crested newt (Fig. 1), is widely distributed from the United Kingdom to northern France, through southern Scandinavia to central Europe, and into a small part of the Balkans. The species is listed on the Habitats Directive of the European Union (92/43/EEC) and is threatened by exposure to fish, habitat loss, and habitat fragmentation (see Jehle et al. 2011; Denoël 2012; Denoël et al. 2013). Thus far, only eight applicable microsatellite loci for *T. cristatus* have been published (Krupa et al. 2002), which might be an insufficient number to reveal consistent results in population genetic analyses (e.g., SPOTG software; Hoban et al. 2013). For the two endemic mountain brook newt species of the genus *Calotriton* (*C. asper*, the Pyrenean brook newt and *C. arnoldi*, the Montseny brook newt, found in the northeastern Iberian Peninsula), no microsatellite loci have been reported thus far. Both species are endemic to comparable small ranges (especially *C. arnoldi*) and are habitat specialists that are adapted to high mountain brooks and have a cryptic life history. *C. asper* is listed as near threatened (NT), and *C. arnoldi* is listed as critically endangered (CR) according to the IUCN Red List v3.1 (http://www.iucnredlist.org/static/categories_criteria_3_1). Therefore, the development of microsatellite loci for these species is an important contribution to efforts to better understand their ecology and evolution and will consequently assist in their conservation. *L. helveticus*, the palmate newt, is distributed throughout Western Europe and is in decline in some parts of Europe due to habitat loss and fragmentation (Denoël and Ficetola 2007). Our study adds additional microsatellite loci to the already existing set of eight loci for this species (Johanet et al. 2009).

### Sampling and DNA extraction

Tissue samples of *T. cristatus* were collected from two different populations in the Kottenforst near Bonn and in the Latumer Bruch in Krefeld (North Rhine-Westphalia, Germany) (Table 1). Tissue samples of *C. asper* were collected from four different sampling sites from the Spanish and French sides of the Pyrenees (Table 1). Samples of *C. arnoldi* were taken from five different locations that were divided into two main sectors (eastern and western) on both sides of the Tordera river valley in the Montseny massif (Spain) that were separated by inhospitable habitat. Tissue samples of *L. helveticus* were collected from five sampling sites in the Larzac plateau (France) (Table 1). Samples were taken by clipping single toes or tail tips, with permission of the local administrative authorities, and stored in 80% ethanol.

**Table 1:** Geographic locations of the sample sites for the different populations. For reasons of conservation, the locations of *Calotriton arnoldi* sample sites are intentionally not listed.

Species	Population	Geographic coordinates
*Triturus cristatus*	Krefeld	6°39′17″E, 51°19′05″N
	Kottenforst	7°3′13″E, 50°40′24″N
*Calotriton asper*	Ibón Perramo	0°29′59″E, 42°38′20″N
	Barranco Valdragás	0°47′38″E, 42°51′29″N
	Ibón d'Acherito	0°42′25″E, 42°52′46″N
	Bassies	1°24′58″E, 42°46′04″N
*Lissotriton helveticus*	Mas d'Aussel	3°19′34″E, 43°58′30″N
	Campels North	3°34′15″E, 43°57′39″N
	Bagnelade	3°21′42″E, 43°51′20″N
	Coulet Northeast	3°32′23″E, 43°49′15″N
	Le Cros Ferme	3°22′10″E, 43°52′11″N

Total genomic DNA was extracted using the sodium-dodecyl-sulfate (SDS)-proteinase K/Phenol–Chloroform extraction method, after which it was stored in Tris-EDTA buffer (10 mmol/L Tris-HCl, 0.1 mmol/L EDTA, pH 8.0) and then used for all subsequent reactions. To start the enrichment procedure for the three distinct target species (i.e., *T. cristatus, L. helveticus,* and *C. asper*) with more or less similar amounts of genomic DNA, we estimated DNA concentrations of different individuals on a 1% agarose gel and selected those with a concentration of 100–200 ng/μL, as suggested by Glenn and Schable (2005).

### Enrichment of microsatellite loci and 454-sequencing

The enrichment protocol followed the selective hybridization method with minor modifications (Zane et al. 2002; Glenn and Schable 2005); and the enrichment procedure was performed separately for each target species. Genomic DNA was digested into approximately 500 bp fragments using Rsa I enzyme and Xmn I to avoid linker dimerization. Double-stranded linkers were annealed to both ends of the fragments to obtain a primer-binding site for subsequent PCR. Linker sequences were as follows: SimpleXL03_U: 5′-AAAACGTGCTGCGGAACT-3′ and SimpleXL03_Lp 5′-pAGTTCCGCAGCACG-3′. PCR was performed in 25 μL reactions to test whether annealing was successful; this PCR product was then used for the next step to increase the concentration of linker-ligated DNA. To capture DNA fragments containing microsatellite loci sequences out of all linker-ligated fragments, 50 μL of Streptavidin M-280 Dynabeads (Invitrogen, Carlsbad, CA, USA) was used. To enrich for tetra-nucleotide motif-bearing DNA fragments, biotinylated oligo probes and linker-ligated DNA fragments were mixed as described by Glenn and Schable (2005). For this step, the following oligo probes were used: (AAGT)_8_, (AGAT)_8_, (ACAT)_8_, (AAAT)_8_, (AACT)_8_, (AAAC)_8_, (AAAG)_6_, (AATC)_6_, (ACAG)_6_, (ACTC)_6_, (ACTG)_6_, (AATG)_6_, and (ACCT)_6_. PCR was performed to recover the microsatellite-enriched DNA fragments (Glenn and Schable 2005). After amplification, all samples were quantified using a Nanodrop spectrophotometer. Afterward, the samples were processed according to the cDNA Rapid Library Preparation Method Manual (Roche, Mannheim, Germany) beginning with step 3.3 and omitting step 3.4. Multiplex Identifier (MID) Adaptors for Rapid Libraries (Roche, Branford, CT) were ligated to the DNA fragments of each sample (*T. cristatus*: MID ACACTACTCGT, MID ACGACACGTAT; *C. asper*: MID ACGAGTAGACT, MID ACGCGTCTAGT; *L. helveticus*: MID ACGTACACACT, MID ACGTACTGTGT). The DNA fragments were cleaned and subsequently quantified using an Agilent 2100 Bioanalyzer. As a final step, the individual samples were combined into a DNA library pool, which was run on an Agilent 2100 Bioanalyzer prior to emulsion PCR and sequencing, as recommended by Roche. The library was not denatured prior to pipetting onto the washed capture beads (step 3.2.8, emPCR Method Manual – Lib-L SV, Roche, Branford, CT, USA). The library was subsequently sequenced on a 454 GS-FLX using Titanium sequencing chemistry.

### Estimation of microsatellite loci success rates for the different species

To estimate the number of PALs based on the numbers of polymorphic loci initially identified, we used steps A through I described below. (A) The PHOBOS software version 3.3.11 (Mayer 2007) was used to assign obtained sequence reads to a target species on the basis of the species-specific MID tags. PHOBOS was also used to identify sequence reads containing noninterrupted stretches of at least ten tetra-nucleotide repeat motifs. (B) Selected sequences were retained by PHOBOS only when the flanking region on each side of the repeat motif was at least 25 bp long. These sequences were then assessed by eye for their general suitability for primer design, that is, sequences with more than five repetitive nucleotides at a stretch were removed. (C) Score values, indicating the quality of each retrieved sequence, were assessed, and sequences with values below 20 (of a maximum score of 40) were discarded from further analysis. (D) We designed primers using the software Primer 3 (version 0.4.0, Rozen and Skaletsky 2000) with default settings (i.e., an optimum primer temperature of 60°C). (E) We tested all primer pairs for amplification success and subsequent degree of polymorphism and heterozygosity in at least 21 individuals. In a first step, a universal M13-tail was attached to the forward primer as a cost-reducing method (Schuelke 2000). (F) Only for the polymorphic microsatellite loci in *C. asper* and *T. cristatus*, we designed primers without the M13-tail but with fluorescence labeling. These primers were tested in a 10 μl of Type-it multiplex PCR (Qiagen) containing 1 μL of DNA for up to 902 individuals per microsatellite locus. Primers were combined in either three (*C. asper*) or two (*T. cristatus*) multiplex mixes, supplemented by one mix of previously published loci for *T. cristatus* (see Krupa et al. 2002). Applied PCR parameters were as follows: (1) an initial *Taq* polymerase activation step of 5 min at 95°C, (2) 30 s at 94°C, (3) 90 s at an annealing temperature of 60°C, (4) 60 s extension at 72°C, (5) steps 2–4 repeated for 30 cycles, and (6) a final extension phase of 30 min at 60°C. PCR products were diluted in 200 μL of water, and 1 μl of diluted product was added to 19 μL of Genescan 500-LIZ size standard (Applied Biosystems) prior to analysis on an ABI 3730 96-capillary automated DNA sequencer. (G) Analysis of the microsatellites was performed using GENEMARKER software (version 1.95, SoftGenetics, State College). Tests for null alleles, deviations from Hardy–Weinberg equilibrium, and linkage disequilibrium were performed using CERVUS (version 3.0.3, Field Genetics Ltd., Marshall et al. 1998). (H) Primer pairs for *C. asper* were also tested for cross-amplification success in *C. arnoldi* using the *C. asper* multiplex mixes with 41 *C. arnoldi* samples originating from two different sectors. Six individuals were tested from sector 1, and 36 individuals were tested from sector 2; PCR conditions were as described for *C. asper*. (I) The estimation of the number of PALs for each species was performed by extrapolating the number of successfully isolated polymorphic loci (SIPL) in relation to the number of tested primer pairs (NTPP). That rate was then used to calculate the number of PALs for sequence reads that passed the criteria described in (B) and (C). Here, an example calculation is provided. We isolated 11 SIPL of 41 NTPP for *T. cristatus,* resulting in a SIPL/NTPP ratio of 26.83%. Extrapolating this ratio to the 107 sequences that passed criteria (B) and (C) resulted in 29 PALs. Thus, the overall success rate (PALs/number of sequences) for *T. cristatus* was 0.15% (see Table 2).

**Table 2:** Number of obtained sequence reads, tested primer pairs (NTPP), successfully isolated polymorphic loci (SIPL) and estimated potentially amplifiable loci PALs as well as corresponding calculated success rates for target species using an enrichment-based 454 next-generation sequencing approach of this study. For comparison, three representative studies using an Illumina next-generation sequencing approach (according to Castoe et al. 2012b) are broken down in comparable units.

454-approach (this study)	Number of sequence reads	Tetra-nucleotides >10 repeats	Tetra-nucleotides >10 repeats + priming sites (>25bp)	NTPP	SIPL	Estimated PALs – success rate (%)
*T. cristatus*	19,562	936	107	41	11	29 (0.15)
*C. asper*	52,075	1083	316	41	20	154 (0.30)
*L. helveticus*	55,626	1434	319	22	15	217 (0.39)

Illumina approach	Number of sequence reads	PAL_FINDER Di, tri, tetra, penta, hexa^2^	Number of tested loci	Number of polymorphic loci	Estimated PALs – success rate (%)
*Cyprinodon julimes/C. pachycephalus*^1^	5 million	1590 loci	48	25 (*C. julimes*)/17 (*C. pachycephalus*)	828 (0.016)/563 (0.0112)
*Leptodea leptodon*^3^	5 million	3905 loci	48	16	1301 (0.026)
*Ambystoma annulatum*^4^	5 million	749 loci^5^	150	22	109 (0.00219)

1 Carson et al. (2013).

2 Castoe et al. (2012a,b).

3 O'Bryhim et al. (2012).

4 Peterman et al. (2013).

5 Note that the study of Peterman et al. (2013) only analyzed tetra- and penta nucleotide motifs.

## Results

The 454-sequencing resulted in a total of 127,263 sequence reads from one quarter run for the enriched libraries of the three species. Sequencing results and success rates of microsatellite loci development are summarized in Table 2. The species classification by MID tag identification of all sequences performed by PHOBOS (Methods, step A) led to 19,562 sequences for *T. cristatus* (15.37% of all sequences)*,* 52,075 sequences for *C. asper* (40.92%), and 55,626 sequences for *L. helveticus* (43.71%). PHOBOS identified 936 sequences (4.78% of all *T. cristatus* reads) containing a noninterrupted stretch of ten or more tetra-nucleotide repeat motifs in *T. cristatus*, 1083 (2.08% of all *C. asper* reads) sequences in *C. asper,* and 1434 (2.58% of all *L. helveticus* reads) sequences in *L. helveticus*. The scan for suitable PCR priming sites (i.e., 25 bp in minimum in each direction) performed in PHOBOS (Methods, step B) resulted in 107 sequences for *T. cristatus*, 316 sequences for *C. asper,* and 319 sequences for *L. helveticus* (Table 2). The assessment for nonrepetitive flanking regions and the exclusion of sequences with score values of poor quality (Methods, steps C and D) led to 41 ordered primer pairs for *T. cristatus*, 41 for *C. asper,* and 22 for *L. helveticus*. In *T. cristatus*, 14 of 41 tested loci were polymorphic (Methods, step E) and were sequentially labeled with fluorescence dyes. In *C. asper*, 21 of the 41 tested loci were polymorphic, while 15 of the 22 tested loci were polymorphic in *L. helveticus*. While the 15 *L. helveticus* loci were analyzed without any additional labeling (Methods, step G), the Type-it multiplex PCR of the *T. cristatus* and *C. asper* loci resulted in the detection of 13 (*T. cristatus*) and 21 (*C. asper*) polymorphic loci (Methods, step F). Validation of these candidate loci (Methods, step G) yielded 11 loci for *T. cristatus*, 20 loci for *C. asper,* and 15 loci for *L. helveticus* (summarized in Tables 3–5). Detailed information on the number of tested individuals per population, the expected and observed heterozygosity, tests for deviations from Hardy–Weinberg equilibrium with a Bonferroni correction, linkage disequilibrium, and report of null alleles is provided for each species in supplementary Tables S1–S3. The test for cross-amplification success of *C. asper* primer pairs in *C. arnoldi* (Methods, step H) resulted in 10 polymorphic loci (Table 4). The SIPL/NTPP ratios (Methods, step I) for *T. cristatus, C. asper,* and *L. helveticus* were 26.83%, 48.78%, and 68.18%, respectively. The adoption of this ratio to calculate the number of PALs resulted in 29 tetra-nucleotide PALs for *T. cristatus*, 154 for *C. asper,* and 217 for *L. helveticus*. The PALs/number of sequences ratio was 0.15% for *T. cristatus*, 0.30% for *C. asper,* and 0.39% for *L. helveticus* (Table 2).

**Table 3:** Characterization of the full set of 17 applied microsatellite loci for *Triturus cristatus*, including the 11 newly developed primer pairs from this study (highlighted in bold) along with six previously published loci (Krupa et al. 2002). Loci are grouped by the multiplex combinations used for amplification. Information on the locus name, primer sequence, direction (F is forward, R is reverse), annealing temperature of the primer for PCRs, microsatellite motif, amplified fragment size range, number of alleles, and labeling dye are provided together with the accession number of the associated GenBank sequence.

Locus name	Primer sequence (5′–3′)	Annealing temp. (°C)	Repeat motif of cloned alleles	Size range of amplification product	Number of alleles	Fluorescence labeling	GenBank accession
Multiplex 1							
Tcri13	F: GTGATGGTTGCCAAGC R: GATCCAAGACACAGAATATTTAG	60°C	(GT)_36_ Interrupted	93–131	10	FAM	AJ292500
Tcri27	F: GATCCACTATAGTGAAAATAAATAATAAG R: CAAGTTAGTATATGATATGCCTTTG	60°C	(GAAA)_27_	241–288	18	FAM	AJ292517
Tcri29	F: CGAGTTGCCCAGACAAG R: GATCACATGCCCATGGA	60°C	(TTTC)_22_(CA)_11_	289–340	11	NED	AJ292505
Tcri35	F: CCAACTGGTATGGCATTG R: GATCACAGAAACTCTGAATATAAGC	60°C	(GAAA)_32_ Interrupted	200–234	10	NED	AJ292490
Tcri36	F: GATCATCTGAATCCCTCTG R: ATACATTCATGACGTTTGG	60°C	(GAAA)_36_ Interrupted	214–320	24	VIC	AJ292491
Tcri46	F: CAAGTTTCCTCTGAAGCCAG R: GTTTCTTGCCTGACAAAGTAATGCTTC	60°C	(TTTC)_23_	253–311	15	PET	AJ292494
Multiplex 2							
**Tc50**	F: GCGGATACATGGTCTTCGTT R: TTCAGTTAAAAGTGTCCTCTGTGG	60°C	(ACTC)_18_	177–268	26	PET	KF442195
**Tc52**	F: GGCTCTTCGACTGAATGGAG R: CGGTCAATTGGTTGTAGCAG	60°C	(ATTG)_17_	190–206	6	VIC	KF442196
**Tc66**	F: CCTTTGTACACCACTGGCAAA R: TGGTCCTATAAAGCCATCTTGG	60°C	(ATCC)_18_	218–250	8	FAM	KF442197
**Tc68b**	F: AAAGTGCACTCTTTCTCTGAAGC R: TGCAAAGTGCATGTGTGACT	60°C	(ATCC)_24_	130–196	13	FAM	KF442198
**Tc70**	F: GGGTTGCAAAGCACCTTAAT R: TACCTGGGTCCTCCTCCAAG	60°C	(ACAT)_14_	216–232	6	VIC	KF442199
**Tc81**	F: TTTAGTCTCTCCGCTCTGCAA R: AGCGGAATCTGCCTTATGGT	60°C	(AATC)_13_	124–160	10	VIC	KF442200
Multiplex 3							
**Tc58**	F: ACAGGCAGTGCGAAAGAAAG R: CTGACCCAAGACCACCTCTC	60°C	(AATC)_7_	200–204	2	NED	KF442201
**Tc69**	F: AGGTAGCCTTCCGCCACTAT R: GCTTGATCCTGGCATGAAAT	60°C	(AGAT)_13_	168–188	6	NED	KF442202
**Tc71**	F: CCGCCAATCAGCAATATTTA R: AGTGGAAGCACCTGCTGAAG	60°C	(ACAT)_11_	179–199	5	PET	KF442203
**Tc74**	F: TCTGTGACATGTCCTGATAGTGAA R: TAGCACCATGAGACCCTCAC	60°C	(AATC)_13_	181–213	9	FAM	KF442204
**Tc85**	F: GTTAGACCTCGCATCTGTTGG R: CCTCAAGACCTGGCTCTACG	60°C	(AATC)_11_	161–169	3	VIC	KF442205

**Table 4:** Characterization of the full set of 20 applied microsatellite loci for *Calotriton asper*. Loci are grouped by multiplex combinations used for amplification. Locus name, primer sequence, direction (F is forward, R is reverse), annealing temperature of the primer for PCRs, microsatellite motif, amplified fragment size range, number of alleles, labeling dye, and GenBank accession number are provided. The number of alleles of *C. asper* microsatellite loci detected in *C. arnoldi* cross-amplification is also provided; polymorphic loci are highlighted in bold.

Locus name	Primer sequence (5′–3′)	Annealing temp. (°C)	Repeat motif of cloned allele	Size range of amplification product	Number of alleles	Number of alleles in *C. arnoldi*	Fluorescence labeling	GenBank accession numbers
Multiplex 1								
Ca1	F: TGGAACAGATGGCGTTGTAA R: TTCCTGCAACCTCCTTGTCT	60°C	(AGAT)_16_	158–170	4	**3**	FAM	KF442206
Ca3	F: CCATGCATTCTTGGAGGTTT R: TTCAAAGGCAGTGTTTCAGG	60°C	(AGAT)_15_	246–288	5	**6**	FAM	KF442207
Ca7	F: ACCCTTACACACCCCAAACC R: GTTCCCTGCATGGCTCTAAA	60°C	(AGAT)_16_	232–264	6	**5**	NED	KF442208
Ca21	F: AGCGTGTGCAGCAGTATCC R: GCAATGTGCCATTCATTACC	60°C	(AGAT)_12_	234–266	7	**5**	VIC	KF442209
Ca22	F: CTTCAGACTGCCGAGTGTTG R: ACCTTGTCACGGTGTAGGAAG	60°C	(AGAT)_13_	140–144	2	**5**	PET	KF442210
Ca24	F: GTGATGTCATGTGCGAGGTC R: GGACCTATGTAAATAGCCCACCT	60°C	(AGAT)_15_	164–180	4	1	NED	KF442211
Us7	F: CTGCACCGATTAATTGCAGA R: CTGCACCACTCGCTCCTC	60°C	(ACAT)_16_	234–242	5	**4**	PET	KF442212
Multiplex 2								
Ca8	F: AGAAGGGAGTCAGGCAGACA R: GGAGGATCAAATGTGTTTGGA	60°C	(AGAT)_13_	174–182	3	2	FAM	KF442213
Ca16	F: GGCAACAATGATGGGTATGC R: ACCGCATGCATGATAGTGCT	60°C	(AGAT)_20_	119–131	4	–	FAM	KF442214
Ca23	F: CGTGCCTGAAACCTATGG R: TTGCTTCACCTCATCCACTG	60°C	(AGAT)_14_	226–266	9	–	PET	KF442215
Ca38	F: CCTGTTAGGTGAAGGTGAGCA R: CTGGTAGCCATGCGCTTTAT	60°C	(AATG)_12_	166–178	3	–	VIC	KF442216
Us2	F: TGGGCTGAAGGATTGAAAAA R: CTCAGCTGCAGTGGTGTGTT	60°C	(AGAT)_17_	242–250	3	**6**	VIC	KF442217
Us3	F: AAGTTTGTAGGTATGCATAATAGCC R: GGAAGTCCAGGCCTGTAGAC	60°C	(AGAT)_16_	184–192	3	**3**	NED	KF442218
Multiplex 3								
Ca5	F: CGTTATTGTCGTGTGGATGG R: TGCTAGTGTAGATCCCTTCATCG	60°C	(ACAT)_10_	218–222	2	–	VIC	KF442219
Ca20	F: CAGCGGTAATACCATCAGGA R: CCACAGATCCTTCTGCAACA	60°C	(AGAT)_15_	200–232	10	–	FAM	KF442220
Ca25	F: CCTTTGTCCCTGTTCAGTGC R: TTTGCAGATGCATTGTGTGA	60°C	(ACAT)_14_	168–172	2	**2**	PET	KF442221
Ca29	F: TCCATAAGCCATTATTGTGTGC R: AGTGCACTGCCTCAGCATGT	60°C	(AATC)_10_	246–258	4	1	PET	KF442222
Ca30	F: TCACACATCATGCAGCTTACC R: GACCCTCATGGGTGTGTAGC	60°C	(AATC)_10_	108–120	3	–	VIC	KF442223
Ca32	F: ACAGGGCAAGAGAGTCAACG R: CAGCCTATTGGCTTGTCAGC	60°C	(ACAG)_10_	148–200	6	**4**	NED	KF442224
Ca35	F: GGCGCTTTACAAGTGCTACC R: CTGCCACAAGGTAGAGGTCA	60°C	(ACTC)_14_	126–166	8	–	FAM	KF442225

**Table 5:** Characterization of the full set of 15 applied microsatellite loci for *Lissotriton helveticus*. Locus name, primer sequence, direction (F is forward, R is reverse), annealing temperature of the primer for PCRs, microsatellite motif, amplified fragment size range, number of alleles and GenBank accession number are provided.

Locus name	Primer sequence (5′–3′)	Annealing temp. (°C)	Repeat motif of cloned allele	Size range of amplification product	Number of alleles	GenBank accession
Lh1	F: CAGCTGCAAGCGACGAAG	60°C	(AGTG)_20_	156–228	10	KF442226
	R: GTTCACACGGATTTGGTTGG					
Lh2	F: TGGCAGGAGAGAGGTTTCAT	60°C	(ATGT)_12_	154–226	6	KF442227
	R: TTGGGACCCTACGGGTAAGT					
Lh6	F: CTGGTGATGTGCTCAGGAGA	60°C	(ATAG)_12_	156–228	9	KF442228
	R: GGAACTGCTTCAATGCCTCT					
Lh7	F: AACATTCCACGCTGTCATCA	60°C	(AATG)_10_	185–189	2	KF442229
	R: GGTCACCGTGCGCTTTATTA					
Lh9	F: GCACATGGTGGAGCTTCAAA	60°C	(AGAT)_10_	172–248	13	KF442230
	R: GACTTGACTGGACCTACTAGTGACA					
Lh12	F: CTCATTACCAAGTCCTGCTTTG	60°C	(AGAT)_19_	164–176	4	KF442231
	R: GGTCGGCTCTTTGTTGCTAA					
Lh13	F: GTCCCCACAGCGTGTGTTAT	60°C	(AACT)_16_	188–208	5	KF442232
	R: CCTCCTGCAGTCCACACC					
Lh14	F: GCAACATCCTCACGTTCTGA	60°C	(AATC)_11_	216–244	5	KF442233
	R: AGCGCATTTAGACCCTCACA					
Lh16	F: TACAGCCTCAGCCATTCACA	60°C	(AATC)_13_	130–142	4	KF442234
	R: TGATGAGATGCGCTCTATAAATAC					
Lh17	F: GCACATGGTGGAGCTTCAAA	60°C	(AGAT)_10_	175–215	4	KF442235
	R: GACTTGACTGGACCTACTAGTGACA					
Lh18	F: GCGCCAGGATACTCTCAAGT	60°C	(ACAT)_11_	137–157	5	KF442236
	R: CAATGGTGAAGGAAGGGCTA					
Lh19	F: CAGTTGTCGCTGGAGGTTG	60°C	(AATC)_10_	191–215	6	KF442237
	R: CTGCCAGTTCCTAGATACACTCA					
Lh44	F: TTTGAGGGACACAACTGATTTT	60°C	(AATC)_15_	231–259	6	KF442238
	R: CTCGCCTTCAGGAGACAACT					
Us4	F: CCATCCTTCCGAGCTCAATA	60°C	(AGAT)_23_	196–204	3	KF442239
	R: TGGGATGGTGTGTCTAAGGTG					
Us9	F: TGGATACCCTGTCAGGTGATTA	60°C	(ATTG)_15_	136–186	6	KF442240
	R: TGCAAGACAGAAGGCTGACA					

## Discussion

### Comparison of success rates of NGS-based microsatellite loci development

For many nonmodel organisms, the de novo development of microsatellite loci has been enormously improved by the implementation of NGS approaches. In the past, the development of microsatellite loci for amphibian species using classic cloning approaches was rather time-consuming and costly, possibly due to their large genomes, which are comparably rich for long repetitive DNA stretches resembling in part microsatellite motifs (e.g., in salamanders; see fig. 1.1 in Steinfartz 2003). Based on our experiences, only the use of enrichment procedures (see Zane et al. 2002) enabled the development of a sufficient number of polymorphic microsatellite loci applicable for genetic studies in various amphibian target species (e.g., Steinfartz et al. 2004; Hauswaldt et al. 2008, 2012; Hendrix et al. 2010). Here, we employed an NGS approach to sequence genomic sublibraries enriched for tetra-nucleotide motifs of three newt species, for which only a limited number (in the case of *T. cristatus* and *L. helveticus*) or no (*C. asper*) loci had previously been available. Although NGS approaches have certainly improved the development of new microsatellite loci, many studies using this approach do not report actual success rates of applicable loci compared with the large number of sequencing reads initially obtained (e.g., Gardner et al. 2011). This might lead to the impression that, by using NGS approaches, very high numbers of new loci can easily be developed. However, the pure occurrence of a microsatellite locus motif in a sequencing read does not guarantee that this locus can be developed into an applicable polymorphic locus for subsequent genetic analyses. Low sequence read quality, motif length (type and number of repeat), and the presence and appropriate length of the surrounding primer region are major factors that can dilute the fraction of potentially amplifiable loci (PALs) enormously. Our aim was therefore to develop high-quality tetra-nucleotide motif-bearing microsatellite loci with a demonstrated utility for subsequent genetic analyses of respective target species. Based on the number of obtained polymorphic loci, we extrapolated species-specific success rates, which were found to be quite low, that is, below one percent (see Table 2).

Although our success rates seem to be unexpected low, they are in line with numerous other studies, from which success rates are reported or can be calculated. Using 454-sequencing technology but no specific enrichment protocol, Castoe et al. (2010) identified 80 tetra-nucleotide PALs (0.06% of total sequencing reads) with more than 10 motif repeats for the copperhead snake (*Agkistrodon contortrix*). In a parallel study in the coral snake (*Micrurus fulvius*), they were able to identify 54 tetra-nucleotide PALs (0.20% of total sequencing reads) with more than 10 motif repeats (Castoe et al. 2012a). Our success rate of 0.15% for *T. cristatus* (29 PALs) was similarly low. In contrast, the estimated 154 PALs for *C. asper* with a success rate of 0.30% and 217 PALs for *L. helveticus* with a success rate of 0.39% were considerably higher. When applying more relaxed comparison criteria between studies, 454-based microsatellite loci development in the meadow viper resulted in only 14 applicable loci out of 37,000 sequence reads (0.037% success rate) and in a success rate of only 0.007% in the Asp viper (Geser et al. 2013) – both studies were performed without prior enrichment of genomic sublibraries. Accordingly, our obtained success rates are comparable quite high and seem to justify the applied enrichment procedure.

There is no doubt that Illumina sequencing is by far more cost-effective than 454-sequencing. Castoe et al. (2012b) suggest that Illumina-based microsatellite loci development is by far more effective than 454-based approaches. In their comparative analysis, they obtain quite high success rates (called discrete PAL rate) ranging from 37–50% for both Illumina- and 454-based approaches, respectively. However, one important drawback of this study was that loci were not specifically tested for final performance and success rates might be therefore strongly overrated. As Illumina sequencing is now commonly applied for microsatellite loci development, we tried to estimate obtained success rates of three studies representative for quite diverse organisms such as fish (Carson et al. 2013), Bivalva (O'Bryhim et al. 2012) and salamanders (Peterman et al. 2013). Although Illumina sequencing resulted in higher number of suitable loci, final success rates were one order smaller than obtained success rates of the combined enrichment-454-sequencing approach (see Table 2). Also here, the enrichment for certain microsatellite loci motifs (e.g., tetra-nucleotides as in our approach) seems to be highly efficient when compared to pure Illumina-based sequencing as evidenced by comparing success rates of our study with the one of Peterman et al. (2013).

### Implications for the use of new microsatellite loci for endangered amphibian species

The newly developed 11 (*T. cristatus*), 20 (*C. asper*), and 15 (*L. helveticus*) polymorphic tetra-nucleotide microsatellite loci will be a tremendous help in furthering our knowledge of the population biology of these locally endangered species, finally building a basis for improved conservation measures. For *T. cristatus*, for example, the set of 19 applicable microsatellite loci will facilitate a more detailed identification of population structure, dispersal, and migration rates across small geographic scales and will even allow for the genetic assignment of single individuals to populations with high credibility. In addition, estimates of effective population sizes of subpopulations or even populations from single ponds can now be identified with much higher resolution. The large number of newly developed applicable loci for *C. asper* sets the foundation for revealing the interesting population biology of this cryptic endemic mountain species, as well as that of its sister species *C. arnoldi*. In particular, new insights into dispersal propensity gained by genetic estimates will be important for elucidating population connectivity, the extent of single populations, the most common reproductive strategies, and how life-history traits relate to individual genotypes. Also, it can be tested whether the unexpected high genetic differentiation of *C. asper* populations based on AFLP markers (Milá et al. 2009) is corroborated by microsatellite loci.

Previous genetic studies in *C. arnoldi* suggested that eastern and western populations belonged to two evolutionary significant units (ESU's; Valbuena-Ureña et al. 2013) and proposed the maintenance of a breeding program for individuals from both units separately. Further genetic studies using microsatellite loci markers to infer the genetic diversity of the species, the current gene flow among population and their possible isolation are urgently needed to evaluate the conservation status more precisely. With the ten *C. asper* microsatellite loci that successfully cross-amplified in *C. arnoldi,* we will be able to study the structure of different populations within the Montseny species range in much greater detail.

For *L. helveticus,* the new loci will be particularly useful for understanding the distribution and success of alternative phenotypes within the species range. Indeed, *L. helveticus* is one of the three European newt species in which facultative paedomorphosis is most regularly reported (Denoël 2007; Denoël et al. 2009). This process results in the retention of larval traits in adults in part of a population, while other individuals metamorphose into the terrestrial morph. Dimorphic populations are particularly common in southern France, where the highest rate of dimorphism is observed in an area that covers only 0.5% of the species range (Denoël 2007). The 23 microsatellite loci now available for this species will be useful for testing evolutionary hypotheses based on gene flow among paedomorphic and metamorphic individuals (see Denoël 2002).

## Conclusion

The use of NGS (454 and Illumina sequencing) strongly facilitated the development of microsatellite loci. However, from most studies, it is unclear how effectively new microsatellite loci can be developed from the large number of sequencing reads obtained from NGS. Our comparative study on three distinct amphibian newt species demonstrates that, despite low overall success rates, the combination of enrichment protocols and NGS can result in considerably higher numbers of polymorphic tetra-nucleotide microsatellite loci. Our study draws a more realistic picture of the efficiency of microsatellite loci development in amphibian species and shows that 454-based microsatellite loci development is still competitive with Illumina-based approaches.

## Supporting Information

**Data S1** Data Accessibility.

**Table S1.** Locus specific characteristics of two populations of *T. cristatus*. Population names, sample sizes (N), and observed (Ho) and expected (He) heterozygosities are provided.

**Table S2.** Locus specific characteristics of up to 4 populations of *C. asper*. Population names, sample sizes (N), and observed (Ho) and expected (He) heterozygosities are provided.

**Table S3.** Locus specific characteristics of 5 populations of *L. helveticus*. Population names, sample sizes (N), and observed (Ho) and expected (He) heterozygosities are provided.

**Table S4.** Locus specific characteristics of two populations of *Calotriton arnoldi*. Population codes, sample sizes (N), and observed (Ho) and expected (He) heterozygosities are provided.
