# Supplementary material for: What remains from a 454 run: estimation of success rates of microsatellite loci development in selected newt species (Calotriton asper, Lissotriton helveticus, and Triturus cristatus) and comparison with Illumina-based approaches
Source: Ecol Evol. 2013 Sep 17;3(11):3947–57. doi: 10.1002/ece3.764 (PMC3810887; doi:10.1002/ece3.764)
Supplement: Supplementary file 1 [file ece30003-3947-SD1.rtf]

Data Accessibility:- New microsatellite loci: GenBank accession numbers xx - xx (will be added upon papers's final acceptance)- Complete sequence reads containing microsatellite motifs filtered by PHOBOS will be uploaded as online supplementary material.
