# Supplementary material for: What remains from a 454 run: estimation of success rates of microsatellite loci development in selected newt species (Calotriton asper, Lissotriton helveticus, and Triturus cristatus) and comparison with Illumina-based approaches
Source: Ecol Evol. 2013 Sep 17;3(11):3947–57. doi: 10.1002/ece3.764 (PMC3810887; doi:10.1002/ece3.764)
Supplement: Supplementary file 2 [file ece30003-3947-SD2.docx]

**Supporting information**

**Table S1:** Locus specific characteristics of two populations of *T. cristatus*. Population names, sample sizes (N), and observed (Ho) and expected (He) heterozygosities are provided. Populations that had significant deviations from Hardy-Weinberg equilibrium following a sequential Bonferroni correction (α = 0.05) are shown with an asterisk (*), and those with a significant frequency of null alleles (*α* = 0*.*05) are denoted by a zero (^0^).

| Locus | Population | N | Ho | He |
| --- | --- | --- | --- | --- |
| Tcri13 | Krefeld | 714 | 0.570 | 0.575 |
|  | Kottenforst | 66 | 0.606 | 0.594 |
| Tcri27 | Krefeld | 728 | 0.823 | 0.830 |
|  | Kottenforst | 59 | 0.831 | 0.862 |
| Tcri29 | Krefeld | 664 | 0.776 | 0.754 |
|  | Kottenforst | 58 | 0.776 | 0.832 |
| Tcri35 | Krefeld | 663 | 0.846 | 0.805 |
|  | Kottenforst | 65 | 0.877 | 0.835 |
| Tcri36 | Krefeld | 730 | 0.830 | 0.845 |
|  | Kottenforst | 65 | 0.692 | 0.715 |
| Tcri46 | Krefeld | 723 | 0.815 | 0.803 |
|  | Kottenforst | 45 | 0.711 | 0.753 |
| Tc50 | Krefeld | 647 | 0.889 | 0.923 |
|  | Kottenforst* | 55 | 0.873 | 0.949 |
| Tc52 | Krefeld | 654 | 0.546 | 0.563 |
|  | Kottenforst | 61 | 0.443 | 0.524 |
| Tc66 | Krefeld | 649 | 0.547 | 0.557 |
|  | Kottenforst | 57 | 0.509 | 0.515 |
| Tc68b | Krefeld | 638 | 0.840 | 0.846 |
|  | Kottenforst | 45 | 0.756 | 0.866 |
| Tc70 | Krefeld | 660 | 0.364 | 0.380 |
|  | Kottenforst | 61 | 0.344 | 0.290 |
| Tc81 | Krefeld | 649 | 0.653 | 0.658 |
|  | Kottenforst | 62 | 0.565 | 0.544 |
| Tc58 | Krefeld | 831 | 0.533 | 0.494 |
|  | Kottenforst | 64 | 0.266 | 0.255 |
| Tc69 | Krefeld | 838 | 0.183 | 0.189 |
|  | Kottenforst^0^ | 64 | 0.344 | 0.381 |
| Tc71 | Krefeld | 837 | 0.466 | 0.494 |
|  | Kottenforst | 62 | 0.548 | 0.533 |
| Tc74 | Krefeld | 834 | 0.621 | 0.583 |
|  | Kottenforst | 64 | 0.563 | 0.487 |
| Tc85 | Krefeld | 836 | 0.057 | 0.058 |
|  | Kottenforst*^0^ | 64 | 0.125 | 0.160 |

**Table S2:** Locus specific characteristics of up to 4 populations of *C. asper.* Population names, sample sizes (N), and observed (Ho) and expected (He) heterozygosities are provided. Populations with significant deviations from Hardy-Weinberg equilibrium following a sequential Bonferroni correction (α = 0.05) are shown with an asterisk (*), and those with a significant frequency of null alleles (*α* = 0*.*05) are denoted by a zero (^0^). Locus specific characteristics for the following loci were only tested in one population: Ca38, Ca5, Ca20, Ca25, Ca29, Ca30, Ca32 and Ca35.

| Locus | Population | N | Ho | He |
| --- | --- | --- | --- | --- |
| Ca1 | Ibón de Perramo* | 48 | 0.146 | 0.138 |
|  | Barranco Valdragás | 39 | 0.821 | 0.790 |
|  | Ibón d‘Acherito | 29 | 0.517 | 0.555 |
|  | Bassies* | 161 | 0.248 | 0.297 |
| Ca3 | Ibón de Perramo ^0^ | 45 | 0.644 | 0.773 |
|  | Bassies*^0^ | 150 | 0.527 | 0.721 |
| Ca7 | Ibón de Perramo | 45 | 0.600 | 0.550 |
|  | Barranco Valdragás | 39 | 0.846 | 0.825 |
|  | Ibón d‘Acherito | 26 | 0.769 | 0.781 |
|  | Bassies | 157 | 0.662 | 0.708 |
| Ca21 | Ibón de Perramo *^0^ | 48 | 0.688 | 0.773 |
|  | Barranco Valdragás | 39 | 0.923 | 0.798 |
|  | Ibón d‘Acherito*^0^ | 21 | 0.095 | 0.814 |
|  | Bassies | 159 | 0.723 | 0.679 |
| Ca22 | Ibón de Perramo *^0^ | 45 | 0.111 | 0.262 |
|  | Barranco Valdragás * | 39 | 0.077 | 0.075 |
|  | Ibón d‘Acherito*^0^ | 27 | 0.370 | 0.307 |
|  | Bassies | 159 | 0.553 | 0.500 |
| Ca24 | Ibón de Perramo ^0^ | 48 | 0.417 | 0.479 |
|  | Bassies | 159 | 0.478 | 0.481 |
| Us7 | Ibón de Perramo *^0^ | 39 | 0.154 | 0.703 |
|  | Barranco Valdragás ^0^ | 38 | 0.763 | 0.877 |
|  | Ibón d‘Acherito*^0^ | 24 | 0.542 | 0.840 |
|  | Bassies | 159 | 0.459 | 0.488 |
| Ca8 | Ibón de Perramo * | 48 | 0.146 | 0.138 |
|  | Barranco Valdragás | 39 | 0.821 | 0.787 |
|  | Ibón d‘Acherito*^0^ | 29 | 0.517 | 0.557 |
|  | Bassies | 153 | 0.412 | 0.416 |
| Ca16 | Ibón de Perramo *^0^ | 29 | 0.172 | 0.607 |
|  | Barranco Valdragás | 39 | 0.923 | 0.863 |
|  | Ibón d‘Acherito*^0^ | 28 | 0.857 | 0.805 |
|  | Bassies | 148 | 0.595 | 0.621 |
| Ca23 | Ibón de Perramo ^0^ | 47 | 0.468 | 0.534 |
|  | Barranco Valdragás ^0^ | 38 | 0.763 | 0.877 |
|  | Ibón d‘Acherito*^0^ | 24 | 0.542 | 0.840 |
|  | Bassies | 146 | 0.808 | 0.801 |
| Ca38 | Ibón d‘Acherito* | 39 | 0.410 | 0.425 |
| Us2 | Ibón de Perramo ^0^ | 48 | 0.667 | 0.775 |
|  | Barranco Valdragás | 39 | 0.949 | 0.802 |
|  | Ibón d‘Acherito*^0^ | 28 | 0.714 | 0.631 |
|  | Bassies | 148 | 0.615 | 0.592 |
| Us3 | Ibón de Perramo | 48 | 0.500 | 0.525 |
|  | Barranco Valdragás | 39 | 0.949 | 0.840 |
|  | Ibón d‘Acherito*^0^ | 30 | 0.800 | 0.812 |
|  | Bassies | 150 | 0.513 | 0.525 |
| Ca5 | Ibón d‘Acherito* | 39 | 0.205 | 0.226 |
| Ca20 | Ibón d‘Acherito*^0^ | 37 | 0.892 | 0.810 |
| Ca25 | Ibón d‘Acherito | 39 | 0.487 | 0.473 |
| Ca29 | Ibón d‘Acherito^0^ | 39 | 0.718 | 0.492 |
| Ca30 | Ibón d‘Acherito | 39 | 0.590 | 0.590 |
| Ca32 | Ibón d‘Acherito^0^ | 39 | 0.538 | 0.589 |
| Ca35 | Ibón d‘Acherito^0^ | 40 | 0.550 | 0.736 |

**Table S3:** Locus specific characteristics of 5 populations of *L. helveticus.* Population names, sample sizes (N), and observed (Ho) and expected (He) heterozygosities are provided. Significant deviations from Hardy-Weinberg equilibrium and null allele frequencies were not assessed because of the small sample size of each population.

| Locus | Population | N | Ho | He |
| --- | --- | --- | --- | --- |
| Lh1 | Mas d’Aussel | 5 | 0.800 | 0.822 |
|  | Campels North | 5 | 0.800 | 0.844 |
|  | Bagnelade | 5 | 0.200 | 0.689 |
|  | Coulet Northeast | 4 | 0.250 | 0.464 |
|  | Le Cros Ferme | 6 | 0.500 | 0.864 |
| Lh2 | Mas d’Aussel | 5 | 0.600 | 0.644 |
|  | Campels North | 6 | 0.333 | 0.439 |
|  | Bagnelade | 5 | 0.400 | 0.533 |
|  | Coulet Northeast | 5 | 0.400 | 0.600 |
|  | Le Cros Ferme | 5 | 0.600 | 0.800 |
| Lh6 | Mas d’Aussel | 5 | 1.000 | 0.844 |
|  | Campels North | 6 | 0.833 | 0.848 |
|  | Bagnelade | 5 | 0.200 | 0.689 |
|  | Coulet Northeast | 5 | 0.600 | 0.800 |
|  | Le Cros Ferme | 6 | 0.833 | 0.909 |
| Lh7 | Mas d’Aussel | 6 | 0.500 | 0.409 |
|  | Campels North | 6 | 0.667 | 0.485 |
|  | Bagnelade | 6 | 0.667 | 0.485 |
|  | Coulet Northeast | 5 | 0.600 | 0.556 |
|  | Le Cros Ferme | 6 | 0.167 | 0.167 |
| Lh9 | Mas d’Aussel | 6 | 0.833 | 0.803 |
|  | Campels North | 6 | 0.667 | 0.879 |
|  | Bagnelade | 6 | 0.667 | 0.818 |
|  | Coulet Northeast | 5 | 0.800 | 0.911 |
|  | Le Cros Ferme | 6 | 0.500 | 0.788 |
| Lh12 | Mas d’Aussel | 4 | 0.500 | 0.429 |
|  | Campels North | 6 | 0.000 | 0.545 |
|  | Bagnelade | 6 | 0.167 | 0.409 |
|  | Coulet Northeast | 4 | 0.250 | 0.250 |
|  | Le Cros Ferme | 6 | 0.333 | 0.318 |
| Lh13 | Mas d’Aussel | 6 | 0.833 | 0.667 |
|  | Campels North | 6 | 0.333 | 0.485 |
|  | Bagnelade | 3 | 0.667 | 0.733 |
|  | Coulet Northeast | 4 | 0.000 | 0.000 |
|  | Le Cros Ferme | 6 | 0.167 | 0.439 |
| Lh14 | Mas d’Aussel | 6 | 0.500 | 0.409 |
|  | Campels North | 6 | 0.333 | 0.530 |
|  | Bagnelade | 5 | 0.600 | 0.467 |
|  | Coulet Northeast | 5 | 0.600 | 0.733 |
|  | Le Cros Ferme | 5 | 0.200 | 0.378 |
| Lh16 | Mas d’Aussel | 6 | 0.167 | 0.621 |
|  | Campels North | 4 | 0.500 | 0.679 |
|  | Bagnelade | 6 | 0.667 | 0..485 |
|  | Coulet Northeast | 6 | 0.667 | 0.758 |
|  | Le Cros Ferme | 6 | 0.167 | 0.591 |
| Lh17 | Mas d’Aussel | 6 | 0.167 | 0.439 |
|  | Campels North | 6 | 0.167 | 0.167 |
|  | Bagnelade | 6 | 0.167 | 0.530 |
|  | Coulet Northeast | 6 | 0.333 | 0.667 |
|  | Le Cros Ferme | 6 | 0.500 | 0.439 |
| Lh18 | Mas d’Aussel | 3 | 0.667 | 0.600 |
|  | Campels North | 4 | 0.000 | 0.000 |
|  | Bagnelade | 5 | 0.200 | 0.733 |
|  | Coulet Northeast | 5 | 0.000 | 0.356 |
|  | Le Cros Ferme | 4 | 0.000 | 0.429 |
| Lh19 | Mas d’Aussel | 5 | 0.800 | 0.689 |
|  | Campels North | 5 | 1.000 | 0.733 |
|  | Bagnelade | 6 | 0.500 | 0.712 |
|  | Coulet Northeast | 4 | 1.000 | 0.821 |
|  | Le Cros Ferme | 6 | 1.000 | 0.864 |
| Lh44 | Mas d’Aussel | 5 | 0.800 | 0.622 |
|  | Campels North | 5 | 0.800 | 0.644 |
|  | Bagnelade | 6 | 0.833 | 0.727 |
|  | Coulet Northeast | 4 | 0.750 | 0.750 |
|  | Le Cros Ferme | 6 | 0.667 | 0.712 |
| Us4 | Mas d’Aussel | 5 | 0.200 | 0.689 |
|  | Campels North | 5 | 0.000 | 0.000 |
|  | Bagnelade | 4 | 0.750 | 0.536 |
|  | Coulet Northeast | 4 | 0.250 | 0.250 |
|  | Le Cros Ferme | 6 | 1.000 | 0.712 |
| Us9 | Mas d’Aussel | 6 | 0.667 | 0.561 |
|  | Campels North | 5 | 0.800 | 0.800 |
|  | Bagnelade | 6 | 0.667 | 0.742 |
|  | Coulet Northeast | 5 | 0.400 | 0.533 |
|  | Le Cros Ferme | 6 | 1.000 | 0.864 |

**Table S4:** Locus specific characteristics of two populations of *Calotriton arnoldi.* Population codes, sample sizes (N), and observed (Ho) and expected (He) heterozygosities are provided. Populations with significant deviations from Hardy-Weinberg equilibrium following a sequential Bonferroni correction (α = 0.05) are shown with an asterisk (*), and those with a significant frequency of null alleles (*α* = 0*.*05) are denoted by a zero (^0^). Characteristics for Ca32 in Sector 1 and Us3 in Sector 2 were not calculated by CERVUS, indicated by a bar (-).

| Locus | Population | N | Ho | He |
| --- | --- | --- | --- | --- |
| Ca1 | Sector 1^0^ | 6 | 0.500 | 0.409 |
|  | Sector 2 | 36 | 0.556 | 0.414 |
| Ca3 | Sector 1^0^ | 4 | 0.750 | 0.750 |
|  | Sector 2 | 34 | 0.618 | 0.547 |
| Ca7 | Sector 1^0^ | 6 | 0.833 | 0.803 |
|  | Sector 2^*0^ | 36 | 0.167 | 0.478 |
| Ca21 | Sector 1 | 6 | 0.833 | 0.727 |
|  | Sector 2 | 36 | 0.444 | 0.430 |
| Ca22 | Sector 1^0^ | 6 | 0.167 | 0.439 |
|  | Sector 2 | 35 | 0.457 | 0.446 |
| Ca25 | Sector 1^*0^ | 5 | 0.200 | 0.200 |
|  | Sector 2^*^ | 34 | 0.235 | 0.211 |
| Ca32 | Sector 1^-^ | 5 | 0.000 | 0.000 |
|  | Sector 2 | 33 | 0.424 | 0.479 |
| Us7 | Sector 1^0^ | 6 | 0.167 | 0.439 |
|  | Sector 2^*^ | 36 | 0.111 | 0.106 |
| Us2 | Sector 1 | 6 | 0.667 | 0.576 |
|  | Sector 2^*0^ | 36 | 0.139 | 0.254 |
| Us3 | Sector 1^0^ | 6 | 0.500 | 0.621 |
|  | Sector 2^-^ | 36 | 0.000 | 0.000 |
